# Multiple Origins of *kdr-type* Resistance in the House Fly, *Musca domestica*

**DOI:** 10.1371/journal.pone.0052761

**Published:** 2012-12-28

**Authors:** Frank D. Rinkevich, Shannon M. Hedtke, Cheryl A. Leichter, Sarah A. Harris, Cathy Su, Seán G. Brady, Vatan Taskin, Xinghui Qiu, Jeffrey G. Scott

**Affiliations:** 1 Department of Entomology, Comstock Hall, Cornell University, Ithaca, New York, United States of America; 2 Department of Entomology, National Museum of Natural History, Smithsonian Institution, Washington, D. C., United States of America; 3 Department of Biology, Faculty of Science, Muğla Sitki Kocman University, Muğla, Turkey; 4 State Key Laboratory of Integrated Management of Pest Insects and Rodents, Institute of Zoology, Chinese Academy of Sciences, Beijing, China; University of Crete, Greece

## Abstract

Insecticide resistance is a model phenotype that can be used to investigate evolutionary processes underlying the spread of alleles across a global landscape, while offering valuable insights into solving the problems that resistant pests present to human health and agriculture. Pyrethroids are one of the most widely used classes of insecticides world-wide and they exert their toxic effects through interactions with the voltage-sensitive sodium channel (Vssc). Specific mutations in *Vssc* (*kdr*, *kdr-his* and *super-kdr*) are known to cause resistance to pyrethroid insecticides in house flies. In order to determine the number of evolutionary origins of *kdr*, *kdr-his* and *super-kdr*, we sequenced a region of *Vssc* from house flies collected in the USA, Turkey and China. Our phylogenetic analysis of *Vssc* unequivocally supports the hypothesis of multiple independent origins of *kdr*, *super-kdr* and *kdr-his* on an unprecedented geographic scale. The implications of these evolutionary processes on pest management are discussed.

## Introduction

Insecticide resistance presents a useful phenotypic trait for studies of evolution because the selective agent is known, the force of selection is strong, and the mutations conferring resistance have often been identified. In addition, the evolution of resistance constrains our abilities to control pest species, resulting in significant economic and health problems. In naïve (i.e., susceptible) populations resistance alleles are rare, with a frequency of 10^−2^ to 10^−13^ [1], [2]. If the resistance allele is completely recessive, then the resistant phenotype will be even rarer. Following insecticide treatment, the frequency of resistance alleles depends on the strength of selection for resistance, the fitness cost of resistance alleles in the absence of insecticide, the initial frequency of resistance alleles, and the likelihood of gene flow among populations [1], [3], [4]. Information about the maintenance and spread of these alleles can improve predictive power for the evolutionary outcome of a particular insecticide regime.

Our understanding of the factors affecting the evolutionary outcome of insecticide use has been greatly enhanced by the development of molecular tools. In particular, sequencing the genes that confer resistance allows us to investigate whether resistance to a specific insecticide or group of insecticides has single or multiple evolutionary origins. In most cases, insecticide resistance appears to have had multiple, independent evolutionary origins that occur in separate geographic regions [5]–[9], although there are a significant number of cases in which resistance appears to have evolved once and then dispersed across the globe [10]–[16].

A major mechanism of resistance to pyrethroids, a commonly used class of insecticides, is target-site insensitivity conferred by mutations in the voltage-sensitive sodium channel gene (*Vssc*) [17]. The first mutation in *Vssc* found to confer pyrethroid resistance (L1014F) is known as *kdr* [18], [19]. In house flies, two other mutations have been identified: L1014H (*kdr-his*) and M918T+L1014F (*super-kdr*). Heterologous expression studies have shown that all three mutations (*kdr*, *kdr-his* and *super-kdr*) result in a sodium channel that is resistant to the effects of pyrethroid insecticides [17], [20]–[24]. For the sake of simplicity, herein we will refer to these as *kdr-type* mutations.

The sequence of the intron that is three base pairs (bp) downstream of the L1014F/H mutation is highly variable in house flies and several other insects. Phylogenetic analyses of *Vssc* haplotypes (including the intron) in house flies suggested multiple evolutionary origins of *kdr-his* and that *super-kdr* was sequentially derived from *kdr* [5]. In the green peach aphid, *Myzus persicae*, a similar analysis indicated multiple independent origins of *kdr* and *super-kdr*, with *super-kdr* again being derived from *kdr* [6]. Multiple origins of pyrethroid resistance have also been found via phylogenetic analysis in *Bemisia tabaci* (silverleaf whitefly), although the mutations conferring resistance differ (L925I and T925V) [7].

In this study, we extensively sampled house fly populations across the USA, Turkey and China. Our phylogenetic analysis of *Vssc* unequivocally supports the hypothesis of multiple independent origins of *kdr*, *super-kdr* and *kdr-his* on an unprecedented geographic scale. In light of our results, we discuss the implications for pest management of selection for and against resistant phenotypes, gene flow among populations, and allelic dominance.

## Materials and Methods

### House Fly Collections

House flies were collected using sweep nets in and around dairy barns at nine locations in the United States (Alachua County FL, Wake County NC, Chemung County NY, Riley County KS, Riverside County CA, Ramsey County MN, Gallatin County MT, Lancaster County NE and Dona Ana County NM). Adult flies were reared for a generation and pupae were mailed to Cornell University. Adult flies of each sex were stored in 70% ethanol at −80°C until used for genotyping. Flies were collected from 15 sites in Turkey and five provinces in China as previously described [14], [15] (Table S1).

### *Vssc* Sequencing

We isolated gDNA from adult female flies collected in the USA as previously described [5]. To assess resistance to pyrethroids, we amplified two fragments of the voltage-sensitive sodium channel (*Vssc*): a 350-bp fragment that included the 1014 codon for evaluating *kdr* and the associated adjacent intron, and a 1.5 kb fragment that included the 918 codon for evaluating *super-kdr* [5]. Sequencing of samples collected in the USA was performed at the Cornell Biotechnology Resource Center. Samples collected from Turkey and China were sequenced as previously described [14], [15].

Electropherograms were inspected and homozygous sequences were compared to previously described haplotypes [5]. Novel haplotypes were named based on the allele present (defined by the deduced amino acids at positions 918 and 1014 of *Vssc*) and then numbered in the order they were discovered. For example, haplotypes that were M918+F1014 were named *kdr1-5*. Those that were M918+H1014 or T918+F1014 were named *kdr-his1-4*, or *superkdr1-3*, respectively. Haplotypes that were M918+L1014 (susceptible) were named *v1-85*. PCR products of the *kdr* region from samples with ambiguous sequences that did not match described haplotypes were cloned into pGEM-T Easy (Promega, Madison WI) and screened and sequenced as previously described [5].

### Phylogenetic Analysis

We used intron+exon sequence from the ∼350 bp region adjacent to the *kdr* mutation to build a phylogenetic tree of sequenced alleles. Sequences were aligned by eye using Mesquite v.2.75 [25]. As there was some uncertainty in the alignment, two matrices were analyzed: one which contained the full matrix (“full”), and another for which all ambiguous cites were excluded (“strict”). Alignments were run in jModelTest v.0.1.1 [26] to determine the best fit model of sequence evolution under the Bayesian Information Criterion. Trees were scored using a branch-length optimization using PhyML [27] as distributed with jModelTest. The maximum-likelihood estimate for each alignment was determined using GARLI v.2.0 for Windows [28], using the best-fit model of sequence evolution, with twenty replicates (search reps = 20). Bootstrap support for each bipartition was determined using GARLI, with only two search replicates for each of 100 bootstrap replicates.

### Parametric Bootstrapping

To assess whether the maximum-likelihood estimate, in which resistant alleles are found throughout the tree, has a statistically significantly higher likelihood than a tree in which there was a single origin of resistance, we used parametric bootstrapping [29]. We performed a phylogenetic analysis on our data, constraining the search to trees in which resistant alleles form a monophyletic grouping (using GARLI [28], with models as above). The statistical distribution of differences in log-likelihood scores under the single origin hypothesis was assessed using simulations on the constrained tree. Simulations were performed using Mesquite [25] with the model parameters estimated by GARLI to produce one hundred replicate data sets. GARLI was once again used to score the best tree and the best constrained tree for each simulated replicate; this generates a distribution of likelihood scores expected if the single-origin hypothesis were correct. We then determined where our observed value fell within this distribution.

## Results

### Haplotypes

We sampled 336 flies from the USA, 73 from Turkey and 46 from China. We identified 103 novel haplotypes, which brings the total number of haplotypes described to more than 120. Some haplotypes contained the same intron sequences and varied only in the coding region of the gene (Table 1). For example, *kdr2*, *super-kdr1*, *kdr-his4* and *v39* all had identical intron sequences, but varied at the codons for amino acids 918 and/or 1014 (Table 1, column B). This was also true for the following pairs of haplotypes: *kdr1* and *super-kdr3*, *kdr4* and *v40*, *kdr5* and *v85*, *kdr-his1* and *v54*, *kdr-his2* and *v41*, *kdr3* and *superkdr2*, and *kdr-his3* and *v42* (Table 1).

**Table 1:** *Vssc* haplotypes found in house fly populations that share the same intron sequence.

1014 Codon	Haplotypes							
	A	B	C	D	E	F	G	H
L		*v39*		*v40*	*v85*	*v54*	*v41*	*v42*
F	*kdr1*	*kdr2*	*kdr3*	*kdr4*	*kdr5*			
H		*kdr-his4*				*kdr-his1*	*kdr-his2*	*kdr-his3*
F+M918T	*super-kdr3*	*super-kdr1*	*super-kdr2*					

Haplotypes in the same column have identical intron sequences. Haplotypes within a column are differentiated solely by the 1014/918 codons. Letters above each column are arbitrary and have no nomenclature significance.

### Phylogenetic Analyses

**Figure 1:**
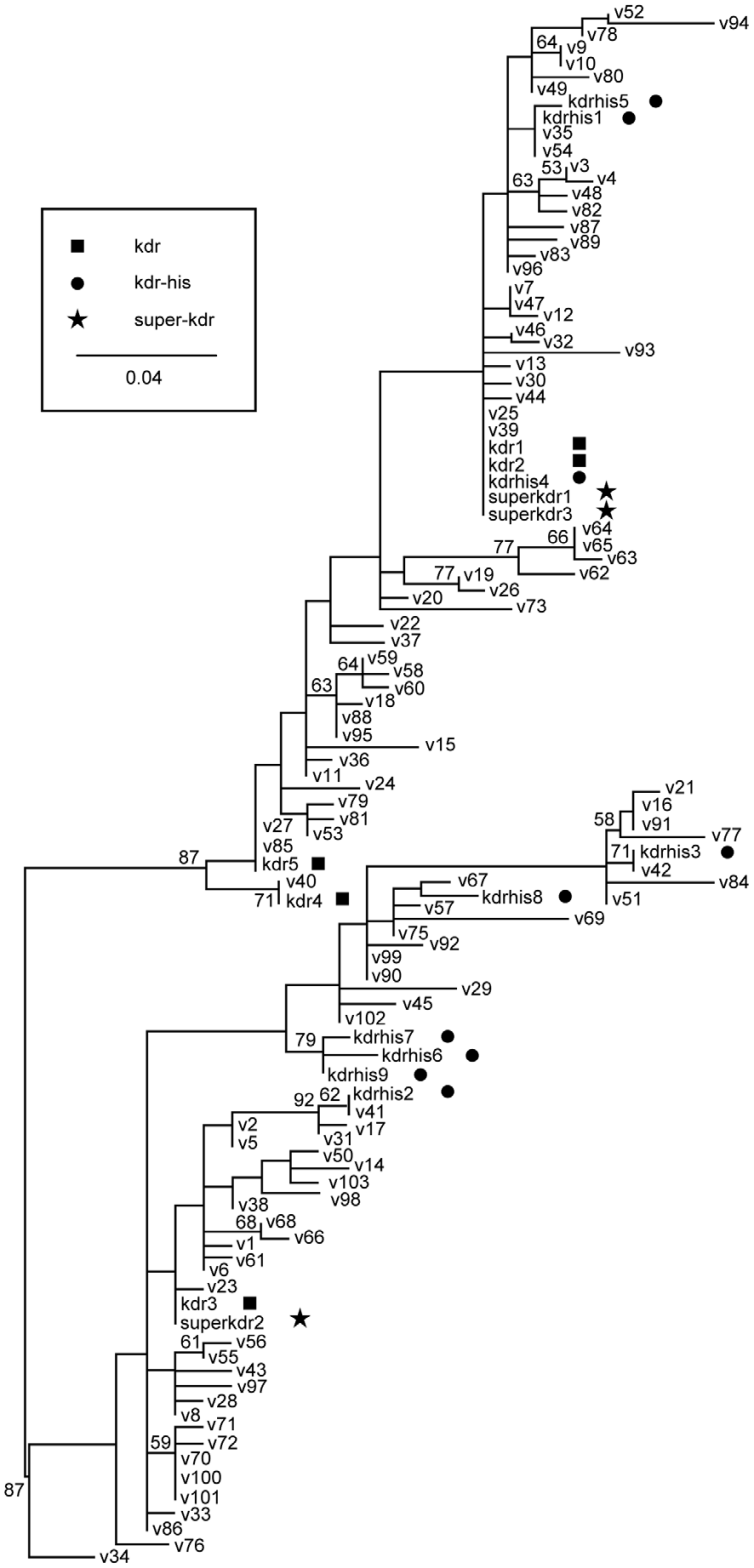
Maximum-likelihood phylogeny of *Vssc* alleles/haplotypes in house flies. Tree is unrooted, and is shown here with a mid-point root for visualization only. Susceptible haplotypes are represented by *v*+number. Numbers at nodes represent the bootstrap values (%); only bootstrap values greater than 50% are shown.

The maximum-likelihood estimate for the full alignment is presented in Figure 1. Analysis of the strict alignment, which eliminated potentially ambiguous sites, resulted in a tree with lower resolution. However, in both trees, there are three independent clades containing *kdr-his* alleles, at least two independent clades containing *kdr* alleles and at least two independent clades containing *super-kdr* alleles, suggesting independent gains or losses of resistance.

### Parametric Bootstrapping

The log-likelihood value of the best tree was −1330.985567, while the log-likelihood of the best tree in which resistant alleles are constrained to a single origin was −1524.707019. The difference in likelihood score, 193.72, falls outside of the distribution of values expected if there were a single origin, and is significant with a p<0.01.

## Discussion

Our analyses strongly support multiple origins of all three *Vssc* alleles: *kdr, kdr-his* and *super-kdr*. Our statistical tests reject the hypothesis of a single origin of resistance to pyrethroids in house flies with strong support (p<0.01). If we make the reasonable assumption that susceptibility is the ancestral condition (since resistance is selected against in the absence of insecticide and is rare in populations not exposed to pyrethroids), then there have been at least two independent origins of *kdr*. Our data further support multiple origins of *kdr-his*, and a sequential progression of *super-kdr* from *kdr* (as in [6]). Although the evolution of *super-kdr* from *kdr* has been found in *M. domestica* and *M. persicae* [5], [6], [30], the M981T mutation has been found without the L1014F mutation in *Aphis gossypii* [31] and *Tetranychus evansi* [32].

Several *kdr* haplotypes have identical intron sequences to susceptible haplotypes (*kdr4* and *v40*; *kdr5* and *v85*), suggesting a single mutational step has recently led from a susceptible to a resistant phenotype. A similar pattern of intron identity between susceptible and resistant phenotypes was observed in *Rdl*, which confers resistance to cyclodienes in the red flour beetle, *Tribolium castaneum* [*33*]. In fact, the ability to find intron haplotypes that have L, F, or H1014 in 6 clusters confirms a thorough sampling effort. In all cases, each *super-kdr* intron haplotype is paired with an identical *kdr* haplotype (Table 1), suggesting that *super-kdr* (M918T+L1014F) likely evolved from an individual house fly with *kdr* (L1014F).

In some cases resistant alleles appear to have evolved once and rapidly spread across a large geographic area [11]. However, in house flies we observe multiple origins of *kdr-type* resistance alleles. Although house flies are capable of dispersing over large geographic areas [34], [35], the genetic structure (based on mitochondrial loci and microsatellites) indicates strong among-population differentiation [36], [37]. The multiple origins of *kdr-type* resistance and the limited geographic distribution of each resistance allele are consistent with independent mutational events in different geographic locations coupled with restricted gene flow and/or a geographic selection mosaic, in which alleles from different populations are selected against.

A geographic mosaic, with limited or rare dispersal coupled with heterogeneous local selection pressures, is suggested by the distribution of *Vssc* haplotypes across the globe (Table S2). House flies are reasonably mobile, being able to fly several km per day. If gene flow drives the distribution of haplotypes, we would expect to detect fewer alleles associated with resistance and greater homogeneity among populations. Instead, our results are consistent with genetically structured populations, as found in previous work [36], [37]. Several resistant alleles have a global distribution (*kdr1*, *kdr2*, *kdr-his4*), but most are restricted to a particular country. For example, *kdr-his2* was detected in the USA only, while *kdr-his5* is restricted to Turkey. Even in the cases where *kdr-his* haplotypes are shared between countries, there are usually very few locations where they are shared, as in the case of *kdr-his4* seen from 10 locations in Turkey, but only from California in the USA. Similarly, susceptible haplotypes *v41* through *v103* were found in only a single country. All *super-kdr* haplotypes were only found in one location (USA). It is probable that *super-kdr* exists in house fly populations in China because it has been detected in a field-collected strain that was selected with deltamethrin [38], but it would appear to be rare [14]. This geographic distribution of resistance haplotypes observed in this study supports multiple, independent origins of resistant *Vssc* alleles.

When attempting to determine the number of origins of a resistance allele, adequate sampling size from a broad geographic range is critical. Previous work on house flies incorrectly concluded there was a single origin of *kdr* [5], likely because there were only two *kdr* haplotypes identified. Our current study, using larger numbers of flies that were sampled from a much wider geographic area, clearly shows that *kdr* has multiple evolutionary origins. Thus, it is important that studies on the evolutionary origins of resistance have a robust number of locations and number of individuals that are sampled.

Based on the cases reported to date, can we identify factors that have led to the multiple origins of resistance in some cases but single origins in others? Clearly the introduction and maintenance of resistant alleles within a population can be affected by multiple factors: the mutation rate from susceptible to resistant alleles (higher frequencies would make multiple origins more likely), the intensity of selection for and against those alleles, and the relative mobility (including movement facilitated by humans) of the pest. Review of the origins of resistance across pest species (Table 2) suggests an important fourth factor: whether the resistance is inherited as a dominant or recessive trait. In all cases where resistance is a recessive trait, there have been multiple origins of resistance. For a fully recessive mutation, resistance is only phenotypically manifest in a homozygote (assuming a diploid organism and an autosomal trait). Such a homozygous resistant individual that dispersed to a new population lacking resistant alleles would produce only heterozygous offspring, with no resistance (assuming fully recessive resistance). In contrast, the arrival of an individual with a dominant resistance mutation would provide resistance to at least half of their offspring (assuming an autosomal locus and diploid organism). If the new population is exposed to pesticide, the mutation can rapidly spread, effectively overcoming the need for the mutation to arise independently in that population. However, the number of cases for which we are able to understand the processes involved in determining the outcome of single or multiple evolutionary origins is limited (Table 2). It would be valuable to determine the number of evolutionary origins for other cases of insecticide resistance, to evaluate the potential connection between the dominance of the alleles underlying these traits and the origin and spread of resistance.

**Table 2:** Resistance mechanisms, patterns of inheritance, evolutionary origins and prevalence of mutations conferring insecticide resistance.

Species	Mechanism	Gene	Pattern of Inheritance	Evolutionary Origin	Citation
*Anopheles gambiae*	Target Site Insensitivity	*Vssc*	Recessive	Multiple	[44]
*Bemisia tabaci*	Target Site Insensitivity	*Vssc*	Recessive	Multiple*	[7]
*Leptinotarsa decemlineata*	Target Site Insensitivity	*Vssc*	Recessive	Multiple	Rinkevich et al, Submitted
*Musca domestica*	Target Site Insensitivity	*Vssc*	Recessive	Multiple	[5] and this paper
*Myzus persicae* and *Myzus nicotinae*	Target Site Insensitivity	*Vssc*	Recessive	Multiple	[6]
*Culex pipiens*	Enhanced Detoxification	*Esterase B*	Completely Dominant	Single	[11]
*Drosophila melanogaster*	Enhanced Detoxification	*CYP6G1*	Dominant	Single	[10]
*Musca domestica*	Enhanced Detoxification	*CYP6D1*	Dominant	Single	[5]
*Myzus persicae*	Enhanced Detoxification	*E4* and *FE4*	Dominant	Single	[12]
*Lucilia cuprina*	Enhanced detoxification	LcαE7 (G137D)	Incompletely Dominant	Single*	[9]
*Lucilia cuprina*	Enhanced detoxification	LcαE7 (T251L)	Incompletely Dominant	Multiple	[9]
*Bemisia tabaci*(B-biotype)	Target Site Insensitivity	*Rdl*	Incompletely Dominant	Single	[8]
*Bemisia tabaci*(non B-biotype)	Target Site Insensitivity	*Rdl*	Incompletely Dominant	Multiple∧	[8]
*Drosophila melanogaster*	Target Site Insensitivity	*Rdl*	Incompletely Dominant	Single	[16]
*Drosophila simulans*	Target Site Insensitivity	*Rdl*	Incompletely Dominant	Multiple	[45]
*Tribolium castaneum*	Target Site Insensitivity	*Rdl*	Incompletely Dominant	Multiple	[33]

* “While phylogenetic analysis of these haplotypes suggests that the Asp137 and Leu251 mutations each arose at least twice, evidence for recombination was detected across the region, therefore single origins for these resistance mutations cannot be ruled out.” [9].

∧The number of species/subspecies of B. tabaci has recently been questioned [46]. If the results above are from different species then findings of multiple origins of resistance need to be interpreted with care.

A confounding factor in understanding the evolution of insecticide resistance is the relative fitness costs of different alleles in the absence of selection. For example, the relative resistance conferred to most pyrethroid insecticides is *super-kdr*>*kdr*>*kdr-his* [5], [39]. However, even in populations where selection pressure is high and resistant individuals predominate, *super-kdr* is rare, and in one population *kdr-his* is the most prevalent allele even though *super-kdr* is also present [40]. This may also be the case in China where *super-kdr* was found in a field-collected strain that was selected in the laboratory with deltamethrin [38], even though in another study *super-kdr* was not detected in field-collected flies and *kdr-his* was the most common allele [14]. Studies in house flies, aphids and mosquitoes have demonstrated a fitness cost for *kdr* in the absence of insecticide [41]–[43], but the relative fitness costs of susceptible, *kdr*, *kdr-his* and *super-kdr* individuals are not well understood. Therefore, an important next step is to further quantify these fitness costs, which will in turn enhance our ability to make predictions about the equilibrium frequency of resistance under alternative pest management strategies.

In conclusion, we have identified a large number of *Vssc* haplotypes that, when subject to phylogenetic analysis, confirms multiple evolutionary origins of *kdr-type* resistance. This study highlights the genetic plasticity that underlies the manifestation of insecticide resistance and has significance for the synthesis of insecticide resistance management strategies. The capacity for convergent evolution of *Vssc* mutations represents a challenge for successful pest management. In particular, the mechanism of resistance and likelihood of gene flow among populations changes our expectations for the maintenance of resistant phenotypes across a species’ global range–and thus, the effects of the selection regime imposed by a particular pesticide application strategy.

## Supporting Information

Table S1 Locations where house flies were collected.
(PDF)

Table S2 Intron haplotypes, GenBank Accession numbers and locations where each haplotype was observed.
(PDF)
