# Supplementary material for: Multiple Origins of kdr-type Resistance in the House Fly, Musca domestica
Source: PLoS One. 2012 Dec 28;7(12):e52761. doi: 10.1371/journal.pone.0052761 (PMC3532202; doi:10.1371/journal.pone.0052761)
Supplement: Table S1 — Locations where house flies were collected. (PDF) [file pone.0052761.s001.pdf]

Supplemental Table 1. Locations from which house flies were collected.

Country	Location	Coordinates	Collection Date	Reference
USA	California, Riverside Co.	33.78/116.8	July 2008	This paper
	Florida, Gilchrist Co.	29.7/82.8	June 2009	This paper
	Kansas, Riley Co.	39.3/96.7	October 2009	This paper
	Minnesota, Ramsey Co.	45.0/93.1	July 2008	This paper
	Montana, Gallatin Co.	45.7/111.1	August 2008	This paper
	Nebraska, Lancaster Co.	40.8/96.7	August 2008	This paper
	New Mexico, Dona Ana Co.	32.3/106.8	July 2008	This paper
	New York, Chemung Co.	42.1/76.8	August 2009	This paper
	North Carolina, Wade Co.	35.9/78.8	July 2009	This paper
Turkey	Adana	37.0/35.3	Summer 2006	[15]
	Afyon	38.8/30.5	“	[15]
	Aydin	37.8/28.5	“	[15]
	Burdur	3.7.7/30.3	“	[15]
	Denizli	37.8/29.1	“	[15]
	Hatay	36.4/36.3	“	[15]
	Isparta	37.7/30.5	“	[15]
	Izmir	38.4/27.1	“	[15]
	Kahramanmaras	37.6/36.9	“	[15]
	Kutahya	39.4/30.0	“	[15]
	Manisa	38.6/27.4	“	[15]
	Mersin	36.8/34.6	“	[15]
	Mugla	37.2/28.4	“	[15]
	Osmaniye	37.1/36.2	“	[15]
	Usak	38.7/29.4	“	[15]
China	Guangdong, Guangzhou	23.1/113.2	Sept/Oct 2009	[14]
	Shanghai, Feng Xian	30.9/121.4	“	[14]
	Shandong, Jinan	36.6/117.9	“	[14]
	Beijing, Chaoyang	40.0/116.4	“	[14]
	Jilin, Changchun	43.9/125.3	“	[14]

Coordinates are given as N/W for locations in the USA and are N/E for locations in Turkey and China.
