# Supplementary material for: Multiple Origins of kdr-type Resistance in the House Fly, Musca domestica
Source: PLoS One. 2012 Dec 28;7(12):e52761. doi: 10.1371/journal.pone.0052761 (PMC3532202; doi:10.1371/journal.pone.0052761)
Supplement: Table S2 — Intron haplotypes, GenBank Accession numbers and locations where each haplotype was observed. (PDF) [file pone.0052761.s002.pdf]

Supplemental Table 2. Intron haplotypes, GenBank Accession numbers and locations where each haplotype was observed.

| Haplotype | Accession # | USA | Turkey | China |
|---|---|---|---|---|
| *kdr1* | AY850260.2 | FL, MN, NC, NE, NY | | Guangdong |
| *kdr2* | AY850261.2 | FL, KS, MN, MT, NE, NY | Adana, Hatay, Isparta, Kahramanmaras, Mersin | Guangdong |
| *kdr3* | HD047549 | CA, KS, MN, NE | | |
| *kdr4* | HD047548 | FL, MT | | |
| *kdr5* | JX139755 | NE | | |
| *kdr-his1* | AY850262.1 | CA, FL, KS, MN, MT, NC, NE, NM, NY | | Guangdong |
| *kdr-his2* | AY850263.2 | FL | | |
| *kdr-his3* | HD04750 | CA, FL, KS, MN, MT, NE, NM | | |
| *kdr-his4* | JX139755 | CA | Afyon, Aydin, Burdur, Denzili, Hatay, Izmir, Kutahya, Manisa, Mersin, Uzak | |
| *kdr-his5* | JX139756 | | Mersin | |
| *kdr-his6* | JX139757 | | | Shandong |
| *kdr-his7* | JX139758 | | | Shandong |
| *kdr-his8* | JX139759 | | | Shandong |
| *kdr-his9* | JX139760 | | Adana, Burdur, Hatay, Isparta, Izmir, Kahramanmaras | Shandong, Shanghai |
| *super-kdr1* | AY850261.2 | NY | | |
| *super-kdr2* | HD047549 | KS, MN, MT, NE | | |
| *super-kdr3* | AY850260 | KS, MN | | |
| v1 | JX174536 | | | |
| v2 | JX174537 | FL | | |
| v3 | AY850270.2 | | | |
| v4 | AY850271.2 | | | |
| v5 | AY850268.3 | NE | Burdur | |
| v6 | AY851288.2 | CA, MN, NE | Aydin, Isparta, Kahramanmaras | |
| v7 | AY850264.2 | CA, MN, MT, NM | Adana, | |
| v8 | AY850265.2 | CA, FL, MN, MT, NE | Manisa | |
| v9 | AY850266.2 | | Mersin | |
| v10 | AY850267.2 | | | |
| v11 | AY850269.2 | | Mugla | |
| v12 | AY850272.2 | | | |
| v13 | AY850273.2 | | | |
| v14 | HD047523 | NY | | |
| v15 | HD047524 | NY | | |
| v16 | HD047525 | MN, MT, NE, NY | Izmir, Osmaniye | Beijing, Shanghai |
| v17 | HD047526 | KS | | |

| | | | | |
|---|---|---|---|---|
| v18 | HD047527 | FL | | |
| v19 | HM047528 | | Mugla | |
| v20 | HD047528 | FL | | |
| v21 | HD047529 | FL | | |
| v22 | HD047530 | FL | | |
| v23 | HD047531 | FL | Mersin, Mugla | |
| v24 | HD047532 | FL | | |
| v25 | HD047533 | FL | | |
| v26 | HD047534 | CA, FL, KS, MT, NE, NM | Osmaniye | |
| v27 | HD047535 | FL | | |
| v28 | HD047536 | FL | Adana, Hatay, Isparta, Kahramanmaras, Osmaniye | |
| v29 | HD047537 | FL | | |
| v30 | HD047538 | FL, NE, NM | Aydin | |
| v31 | HD047539 | CA, FL, NE, NM | Izmir | |
| v32 | HD047540 | FL | | |
| v33 | HD047541 | CA, KS, MN, MT, NE, NM | | Guangdong |
| v34 | HD047542 | KS, MN | | |
| v35 | HD047543 | KS | | |
| v36 | HD047544 | KS, NE | | |
| v37 | HD047545 | KS | Mugla | |
| v38 | HM047547 | | Aydin, Isparta | Jilin |
| v39 | AY850261 | CA, FL, KS, MN, MT, NE, NM | Adana, Aydin, Burdur, Hatay, Izmir | Guangdong |
| v40 | HD047546 | FL | Afyon, Osmaniye | |
| v41 | AY850263 | KS | | |
| v42 | HD047547 | CA, KS, NE, NM | | |
| v43 | JX174538 | NE | | |
| v44 | JX174539 | | | |
| v45 | JX174540 | | Kahramanmaras | |
| v46 | JX174541 | | Adana | |
| v47 | JX174542 | | Mugla, | |
| v48 | JX174543 | | | |
| v49 | JX174544 | | Hatay, Isparta, Mersin | |
| v50 | JX174545 | | Isparta | |
| v51 | JX174546 | | Burdur | |
| v52 | JX174547 | | Afyon | |
| v53 | JX174548 | | Adana, Denizli | |
| v54 | JX174549 | | | |
| v55 | JX174550 | | | Guangdong |
| v56 | JX174551 | | | Guangdong |
| v57 | JX174552 | | | Beijing |
| v58 | JX174553 | | | Beijing |
| v59 | JX174554 | | | Beijing |
| v60 | JX174555 | | | Beijing |
| v61 | JX174556 | | | Jilin |
| v62 | JX174557 | | | Jilin |
| v63 | JX174558 | | | Jilin |
| v64 | JX174559 | | | Jilin |

| | | | | |
|---|---|---|---|---|
| v65 | JX174560 | | | |
| v66 | JX174561 | | | Shandong |
| v67 | JX174562 | | | Shandong |
| v68 | JX174563 | | | Shandong |
| v69 | JX174564 | | | Shanghai |
| v70 | JX174565 | | | Shanghai |
| v71 | JX174566 | | | Shanghai |
| v72 | JX174567 | | | Shanghai |
| v73 | JX174568 | KS | | |
| v74 | JX174569 | CA. MT | | |
| v75 | JX174570 | CA, MT | | |
| v76 | JX174571 | CA, MT, NM | | |
| v77 | JX174572 | MT | | |
| v78 | JX174573 | CA, NE, NM | | |
| v79 | JX174574 | NE | | |
| v80 | JX174575 | CA, MN, NE, NM | | |
| v81 | JX174576 | | Aydin, Izmir, Manisa | |
| v82 | JX174577 | | | |
| v83 | JX174578 | | | |
| v84 | JX174579 | | Burdur | |
| v85 | JX174580 | | Manisa | |
| v86 | JX174581 | | Manisa, Mersin | |
| v87 | JX174582 | | Manisa | |
| v88 | JX174583 | | Adana | |
| v89 | JX174584 | | Adana | |
| v90 | JX174585 | | Hatay, Uzak | |
| v91 | JX174586 | | Hatay | |
| v92 | JX174587 | | Isparta | |
| v93 | JX174588 | | Mersin | |
| v94 | JX174589 | | Isparta | |
| v95 | JX174590 | | Denzili | |
| v96 | JX174591 | | Denzili | |
| v97 | JX174592 | | Denzili | |
| v98 | JX174593 | | Uzak | |
| v99 | JX174594 | | Uzak | |
| v100 | JX174595 | | Denzili | |
| v101 | JX174596 | | Denzili | |
| v102 | JX174597 | | Denzili | |
| v103 | JX174598 | | Denzili | |
